# Recurrence Rate and Risk Factors for Apical Prolapse After Sacrospinous Ligament Fixation: A Prospective Cohort Study

**DOI:** 10.7759/cureus.106420

**Published:** 2026-04-04

**Authors:** Musa Kayondo, Rogers Kajabwangu, Onesmus Byamukama, Brenda Ainomugisha, Kalyebara Paul Kato, Leevan Tibaijuka, Henry M Lugobe, Verena Geissbuehler

**Affiliations:** 1 Obstetrics and Gynaecology, Mbarara University of Science and Technology, Mbarara, UGA; 2 Obstetrics and Gynecology, Mbarara University of Science and Technology, Mbarara, UGA; 3 Obstetrics and Gynaecology, University of Basel, Basel, CHE

**Keywords:** apical prolapse, recurrence, risk factors, sacrospinous ligament fixation, uganda

## Abstract

Objective: We aimed to determine the one-year recurrence rate of apical prolapse following sacrospinous ligament fixation and to identify clinical and surgical risk factors associated with recurrence in a low-resource setting.

Methods: This was a prospective cohort study of women who underwent sacrospinous ligament fixation as part of surgery for apical prolapse at the urogynecology unit of Mbarara Regional Referral Hospital (MRRH) in Uganda. The surgeries performed included vaginal hysterectomy with sacrospinous ligament vault fixation, sacrospinous ligament hysteropexy, and sacrospinous ligament vault fixation for those with post-hysterectomy vaginal vault prolapse. Concomitant procedures such as anterior or posterior repair, or both, were performed for women with prolapse in other compartments. The women were followed up for a period of one year post-surgery. Recurrence was assessed with the women in lithotomy position under maximum strain using the Pelvic Organ Quantification (POP-Q) system. Recurrence was defined as apical prolapse of ≥POP-Q stage II. Multivariable log-binomial regression was performed to determine risk factors for recurrence.

Results: A total of 123 participants were enrolled in this study, of which 111 (90.2%) completed follow-up. The mean age was 53.1 (SD ±13.6) years. The majority of apical prolapse was uterine (94.6%) and classified as POP-Q stage III (58.6%). The recurrence rate was 19.8% (22/111, 95% CI: 13.4-28.4). Risk factors for apical prolapse recurrence included body mass index (BMI) >25 kg/m^2^ (relative risk (RR) = 6.02; 95% confidence interval (CI): 2.05-17.67; p* = *0.001) and post-operative complications (RR = 19.87; 95% CI: 5.77-68.47; p *< *0.001). Undergoing vaginal hysterectomy as part of the prolapse surgery was found to be protective (RR = 0.09; 95% CI: 0.03-0.25; p *< *0.001).

Conclusions: Apical prolapse recurrence after sacrospinous ligament fixation is common in this setting. To reduce the risk of recurrence, management protocols should prioritize prevention and timely management of postoperative complications, and counseling for weight optimization. Furthermore, vaginal hysterectomy should be considered in uterine prolapse where uterine sparing surgery is not required.

## Introduction

Pelvic organ prolapse (POP) is an important public health issue that negatively impacts the quality of life of women, especially in developing countries [1,2]. POP affects women globally, with prevalence estimates ranging from 3% to 11% by self-report (questionnaires) and up to 32-98% on physical examination [3]. In Uganda, 27.5% of women attending gynecology outpatient clinics have been diagnosed with POP [4]. In rural southwestern Uganda, women commonly have high parity and engage in physically demanding labor, which may contribute to both the severity of prolapse and the risk of recurrence following surgical repair [5].

The apical vaginal compartment, comprising the upper third of the vagina and the uterus, is supported on the pelvic wall by the caudal portion of the cardinal and uterosacral ligaments (level-one support). Weakness in the level-one support often leads to apical compartment prolapse [6]. The apical compartment is a very important aspect of the vagina as it contributes to the support of all other vaginal compartments. Therefore, surgery for treatment of apical prolapse should be sufficient since it prevents prolapse of the other compartments [7,8].

Several surgical techniques restore apical support by anchoring the vaginal apex to different ligaments, including the uterosacral, sacrospinous, iliococcygeal, and anterior longitudinal ligament of the sacrum [9]. Sacrospinous ligament fixation is a common procedure for apical suspension [10,11]. This procedure, first described by Richter in 1968 [12], involves suspension of the vaginal vault to the sacrospinous ligament after vaginal hysterectomy or in patients with vault prolapse following hysterectomy. Similarly, in 1989, Richardson et al. reported on sacrospinous ligament hysteropexy, a method of suspending the uterus in women with significant uterine descent where the uterus needed to be preserved [13].

Studies have shown sacrospinous ligament fixation as a safe, low-cost, and effective procedure in the management of apical prolapse [9,14-17]. Despite its effectiveness, recurrence rates after sacrospinous fixation vary widely (17-42%), influenced by patient and surgical factors, such as younger age <60 years, high body mass index (BMI), advanced preoperative POP-Q stage, and smoking [18-20].

However, most evidence on recurrence and risk factors comes from high-income settings. Data from low-resource settings, where sacrospinous ligament fixation is frequently performed for apical suspension, remain scarce. This study, therefore, aimed to determine the one-year recurrence rate of apical prolapse following sacrospinous ligament fixation and to identify clinical and surgical risk factors associated with recurrence in a low-resource setting.

This study was previously presented as a meeting abstract at the 49th Annual Meeting of the International Urogynecological Association (IUGA), held in Singapore, June 19-22, 2024.

## Materials and methods

Study setting

We conducted the study at the Urogynecology Unit of Mbarara Regional Referral Hospital (MRRH), a tertiary referral hospital in Southwestern Uganda, from January 2022 to December 2023.

Study design

This was a prospective cohort study of women who underwent sacrospinous ligament fixation as part of surgery for apical prolapse and were followed up for one year post-surgery.

Study population and enrollment

We enrolled women diagnosed with symptomatic apical prolapse. Participants were considered to have apical prolapse if they had either uterine prolapse or vaginal vault prolapse as part of the POP. Staging of the apical prolapse was done using the Pelvic Organ Prolapse Quantification (POP-Q) system validated by the International Continence Society (ICS) into stages I, II, III, and IV in the lithotomy position under maximal strain [21,22]. We only included women with stage III and IV apical prolapse. Informed consent was obtained from all eligible participants.

Surgical technique

The participants underwent sacrospinous ligament fixation as part of surgery for the management of apical prolapse after obtaining informed consent. All patients received a single-dose preoperative antibiotic just before commencement of surgery. The surgeries were performed under spinal anesthesia with the participant in lithotomy position, and the surgeries were done by a team of subspecialty surgeons (certified urogynecologists), as part of routine management of POP at the hospital. Participants who did not desire uterine preservation and had provided prior consent underwent vaginal hysterectomy, followed by sacrospinous ligament vault fixation. This procedure was also performed in patients presenting with vault prolapse after a previous hysterectomy. For participants who wished to preserve the uterus, sacrospinous hysteropexy was carried out. Whether the uterus was preserved or not, concomitant procedures such as anterior or posterior wall repair, or both, were performed in participants with prolapse in other compartments. Sacrospinous ligament fixation procedure, as described by Randall et al. [23], was performed through an incision in the posterior vaginal wall to open into the rectovaginal space and further advancing into the pararectal space by a combination of blunt and sharp dissection. Using a combination of two Harney and one diver’s retractor, the sacrospinous ligament was exposed by retracting the rectum medially and cephalad. A non-absorbable suture of Prolene 0 (Ethicon, Sommerville, New Jersey, USA) on a round-bodied needle was inserted into the ligament approximately 2 cm medial to the ischial spine and, subsequently, inserted through the vaginal apex for participants that underwent colpopexy or the posterior aspect of the cervix for those that underwent hysteropexy. The procedure was done bilaterally. The apex was then fixed onto the sacrospinous ligament by tying the sutures after closing the posterior vaginal wall incision with Vicryl 2/0 suture. At the end of the operation, an indwelling urinary catheter was inserted in all the participants. This was removed the morning after the operation, except for participants who had additional anterior repair, where it was removed after three days. Other aspects of the postoperative care, like antibiotics, laxatives, fluid administration, and analgesia, were given to all participants, which is the routine protocol in the unit.

Data collection and study variables

A data capture tool was used to collect information on the baseline characteristics of the study participants, intraoperative findings, and the post-operative follow-up information. The baseline characteristics included 1) sociodemographic characteristics (age, BMI, marital status, and occupation), 2) gynecological history (parity and menopausal status), 3) type of apical prolapse (uterine and vaginal vault), 4) concomitant prolapse in other compartments (anterior and posterior wall), and 5) preoperative POP-Q stage (III and IV). The intraoperative information that was collected included 1) primary prolapse procedure performed (vaginal hysterectomy with sacrospinous ligament fixation, and sacrospinous hysteropexy), 2) additional prolapse surgeries (anterior and posterior repair), 3) nature of sacrospinous fixation (primary and repeat), and 3) cadre of surgeon (fellow and urogynecologist). Postoperative information collected included 1) postoperative complications (hematoma in the pararectal space and vaginal cuff infection) and 2) days spent on the ward after surgery. A participant was considered to have vaginal cuff infection if she had increasing lower abdominal pain, purulent vaginal discharge, and a tender surgical site on physical examination [24]. Hematoma in the pararectal space was suspected if a participant had vaginal and buttock pain plus a mass in the lateral vaginal aspect on digital rectal examination. The data capture form was filled out by the trained research assistants (nurses and surgeons).

Follow-up of the participants

After discharge from the hospital, the participants were followed up for one year after surgery to assess for recurrence of apical prolapse. Participants were contacted through a phone call to remind them of their scheduled follow-up visit. Those who could not be reached on the phone were traced using the contact of their next of kin. At each follow-up visit, a pelvic examination with the participant in lithotomy position under maximal strain was done to assess for recurrence. This assessment was done by a trained research assistant (a gynecologist) who was not part of the initial surgical team. A participant was considered to have a recurrence if she had a bulge ≥ POP-Q stage II in the apical compartment on maximum straining [25]. 

Statistical analysis

This study was conducted as a nested analysis within a larger prospective cohort, and all eligible participants undergoing sacrospinous ligament fixation during the study period were consecutively included. As such, a formal sample size calculation was not performed. Data were entered into RedCap and exported to Stata 17 (StataCorp, LLP, College Station, TX, USA) for analysis. Categorical data were presented as frequencies. The recurrence rate was determined by dividing the number of women who had recurrence in the apical compartment at one year by the total number of women who completed the follow-up and expressed as a percentage. To determine the risk factors for recurrence, univariable and multivariable analyses were performed using log binomial regression analysis. Risk ratios (RR) and their corresponding 95% confidence intervals (CI) were reported as the measures of association. Factors with a p-value <0.2 at univariable analysis were included in the final multivariable model to determine the adjusted risk factors for recurrence. A p-value < 0.05 was considered statistically significant.

Ethical considerations

The study was a sub-study of a bigger study on pelvic floor disorders that received approval from the Ethics Committee of Mbarara University of Science and Technology (reference number: 17/08-18) and Uganda National Council for Science and Technology (number: HS368ES). We informed the participants of the study objectives and recruited only those who gave written consent. Confidentiality was observed during all the interviews and examinations.

## Results

**Figure 1:**
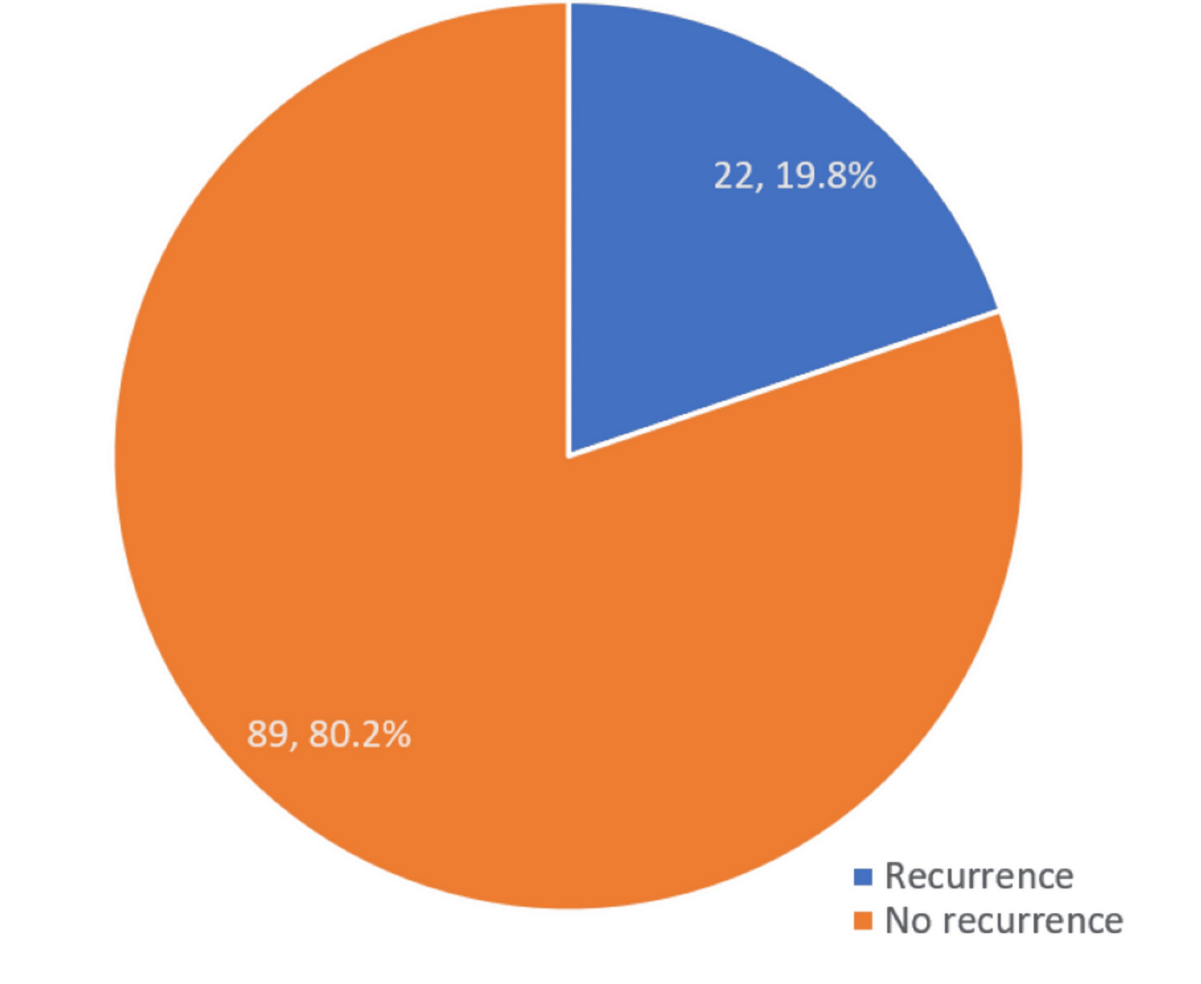
Apical prolapse recurrence at one-year follow-up

A total of 123 participants were enrolled in this study, of which 111 (90.2%) completed the follow-up period of one year. Therefore, the results being reported are for 111 participants. Of the 111 participants who completed the follow-up period, the cumulative number of women with recurrence in the apical compartment was 22. Therefore, the recurrence rate was 19.8% (95% CI: 13.4-28.4) (Figure 1).

The baseline characteristics of the study participants are shown in Table 1. The mean age of the participants was 53.1 (SD ±13.6) years. Most participants were peasant farmers (n = 91, 82%) and multiparas (n = 105, 94.6%). Uterine prolapse was the most common type of apical prolapse (n = 105, 94.6%), and the majority of participants had a preoperative POP-Q stage III (n = 65, 58.6%). All operations were performed under spinal anesthesia, and most were primary surgeries (n = 101, 91.0%). The type of surgery differed significantly by recurrence status (p < 0.001). Recurrence was most frequent among women who underwent sacrospinous hysteropexy (45.5%), compared to those who underwent hysterectomy with sacrospinous ligament fixation (13.6%), while no recurrences were observed among women who underwent sacrospinous ligament fixation alone due to vault prolapse following previous hysterectomy. Additional surgeries for prolapse in other compartments were performed in 89 (80.2%) participants, and the most common was anterior repair (n = 70, 63.1%). Postoperative complications were encountered in only eight (7.2%) participants, and the most common was hematoma in the pararectal space (5, 4.5%). There was no difference in the baseline characteristics in the two groups except in those who had vaginal hysterectomy, sacrospinous hysteropexy, and those who suffered postoperative complications.

**Table 1:** Demographic, clinical and perioperative characteristics of the study participants by recurrence status Data are presented as mean ± standard deviation (SD) for continuous variables and as frequencies with percentages (n (%)) for categorical variables. Differences between groups were assessed using the independent t-test for continuous variables and the chi-square (χ²) test for categorical variables. Statistical significance was set at p < 0.05. SD: standard deviation; POP-Q: Pelvic Organ Prolapse Quantification system; BMI: body mass index; χ²: chi-square; SSLVF: sacrospinous ligament vault fixation

Characteristics	Category		Recurrence		p-value
		Total (N = 111)	Yes (N = 22)	No (N = 89)	
		n (%)	n (%)	n (%)	
Mean age in years ±SD		53.1±13.6	53.4±16.5	53.0±12.9	0.91
Age categories (years)					0.66
	<50	45 (40.5)	8 (36.4)	37 (41.6)	
	≥50	66 (59.5)	14 (63.6)	52 (58.4)	
Marital status					0.75
	Married	98 (88.3)	19 (86.4)	79 (88.8)	
	Unmarried	13 (11.7)	3 (13.6)	10 (11.2)	
Occupation					0.21
	Peasant	91 (82.0)	16 (72.7)	75 (84.3)	
	Skilled professional	20 (18.0)	6 (27.3)	14 (15.7)	
Parity					0.39
	Primiparous (I)	6 (5.4)	2 (9.1)	4 (4.5)	
	Multiparous (≥II)	105 (94.6)	20 (90.9)	85 (95.5)	
Body mass index (kg/m^2^)					0.24
	<18.5	8 (7.2)	1 (4.5)	7 (7.9)	
	18.5-<25	66 (59.5)	9 (41)	57 (64.0)	
	>25	37 (33.3)	12 (54.5)	25 (28.1)	
Reached menopause	Yes	62 (55.9)	12 (54.5)	50 (56.2)	0.89
Type of apical prolapse					0.21
	Uterine	105 (94.6)	22 (100.0)	83 (93.3)	
	Vault	6 (5.4)	0 (0.0)	6 (6.7)	
Pre-op stage of apical prolapse (POP-Q)					0.59
	III	65 (58.6)	14 (63.6)	51 (57.3)	
	IV	46 (41.4)	8 (36.4)	38 (42.7)	
Concomitant compartment prolapse					0.34
	Anterior and posterior	15 (13.5)	4 (18.2)	11 (12.4)	
	Anterior only	70 (63.1)	13 (59.1)	57 (64.0)	
	Posterior only	4 (3.6)	2 (9.1)	2 (2.2)	
	No	22 (19.8)	3 (13.6)	19 (21.3)	
Type of surgery					0.41
	Repeat	10 (9.0)	1 (4.5)	9 (10.1)	
	Primary	101 (91.0)	21 (95.5)	80 (89.9)	
Main surgery performed					<0.001*
	SSLVF alone	6 (5.4)	0 (0.0)	6 (6.7)	
	Hysterectomy +SSLVF	88 (79.3)	12 (54.5)	76 (85.4)	
	Sacrospinous hysteropexy	17 (15.3)	10 (45.5)	7 (7.9)	
Additional surgeries (repairs)					0.52
	Anterior + posterior	16 (14.4)	5 (22.7)	11 (12.4)	
	Anterior repair	70 (63.1)	13 (59.1)	57 (64.0)	
	Posterior	3 (2.7)	1 (4.5)	2 (2.3)	
	None	22 (19.8)	3 (13.6)	19 (21.3)	
Cadre of surgeon					0.67
	Fellow	56 (50.5)	12 (54.5)	44 (49.4)	
	Urogynecologist	55 (49.5)	10 (45.5)	45 (50.6)	
Type of post-op complication					<0.001*
	Hematoma	5 (4.5)	4 (18.2)	1 (1.1)	
	Vaginal cuff infection	3 (2.7)	3 (13.6)	0 (0.0)	
	No complication	103 (92.8)	15 (68.2)	88 (98.9)	

The risk factors for recurrence of apical prolapse after sacrospinous ligament fixation are shown in Table 2. We found that women with BMI > 25 kg/m² (RR = 6.02; 95% CI: 2.05-17.67; p = 0.001) and women who had postoperative complications (RR = 19.87; 95% CI: 5.77-68.47; p < 0.001) were at risk of recurrence of apical prolapse one year after sacrospinous ligament fixation. However, women who had a vaginal hysterectomy as part of the prolapse surgery were less likely to get apical recurrence after sacrospinous ligament fixation (RR = 0.09; 95% CI: 0.03-0.25; p < 0.001).

**Table 2:** Risk factors for recurrence of apical prolapse after sacrospinous ligament fixation among Ugandan women RR: risk ratio; CI: confidence interval, *p < 0.05

Variable		Recurrence		Multivariable analysis	
		Yes	No	Adjusted RR (95% CI)	P-value
		N = 22	N = 89		
		n (%)	n (%)		
Age (years)					
	<50	8 (36.4)	37 (41.6)	1	
	≥50	14 (63.6)	52 (58.4)	1.61 (0.75, 3.48)	0.225
Parity					
	Primiparous (1)	2 (9.1)	4 (4.5)	1	
	Multiparous (≥2)	20 (90.9)	85 (95.5)	0.54 (0.17, 1.67)	0.284
Body mass index (kg/m^2^)					
	Underweight <18.5	1 (4.5)	7 (7.9)	3.33 (0.65, 17.05)	0.148
	Normal (18.5-<25)	9 (41)	57 (64.0)	1	
	Overweight/obese >25	12 (54.5)	25 (28.1)	6.02(2.05, 17.67)	0.001*
Pre-op stage of apical prolapse (POP-Q)					
	III	14 (63.6)	51 (57.3)	1	
	IV	8 (36.4)	38 (42.7)	1.41 (0.77, 2.58)	0.268
Type of surgery					
	Repeat	1 (4.5)	9 (10.1)	1.35(0.64, 1.90)	0.222
	Primary	21 (95.5)	80 (89.9)	1	
Vaginal hysterectomy done					
	No	10 (45.5)	13 (14.6)	1	
	Yes	12 (54.5)	76 (85.4)	0.09 (0.03, 0.25)	<0.001*
Cadre of surgeon					
	Fellow	12 (54.5)	44 (49.4)	1.84 (0.79, 4.27)	0.154
	Urogynecologist	10 (45.5)	45 (50.6)	1	
Postoperative complications					
	None	15 (68.2)	88 (98.9)	1	
	Yes	7 (31.8%)	1 (1.1%)	19.87 (5.77, 68.47)	<0.001*

## Discussion

This hospital-based prospective cohort study determined the recurrence rate of apical prolapse at one year and risk factors for recurrence at MRRH in southwestern Uganda. We found a recurrence rate of 19.8% at one year after surgery. Risk factors for recurrence were BMI >25 kg/m² and postoperative complications. Performing a vaginal hysterectomy before the fixation was protective against recurrence.

The recurrence rate in this study is similar to what was reported by Dietz V et al., i.e., 17% in a follow-up study of one year [26]. However, it is lower than that found in other studies [16,17,19] where rates of 42%, 35%, and 27% were reported. The contrast could be due to the difference in the follow-up period. In all the contrasting studies, the follow-up period was longer (five years) compared to ours of one year. Probably the fixation weakens with time due to tissue remodeling and stretching, leading to the higher recurrence rate seen in studies with longer follow-up periods. However, Favre-Inhofer did not find any statistical difference between the recurrence rate in patients followed up for <5 years and those followed up for >5 years [16].

The recurrence rate is comparable to that of other techniques of apical compartment suspension [27,28]. Pedersen found an apical prolapse recurrence rate of 19% in a retrospective study of women who underwent high uterosacral ligament suspension [28]. A Cochrane review by Maher et al. (2019) did not find any difference between sacrospinous ligament fixation and abdominal sacrocolpopexy in subjective recurrence [27]. 

In this study, obesity/overweight was found to be one of the risk factors for apical prolapse recurrence after sacrospinous ligament fixation. This is similar to other studies [26, 29]. Obesity may lead to recurrence through an increase in intra-abdominal pressure that exerts persistent strain and stretch on the sacrospinous ligament, other support tissues like fascia, and the fixation sutures [20]. While BMI >25 kg/m² is an established threshold for overweight using international classifications, its interpretation in our predominantly rural population warrants caution. A BMI just above this cutoff may not reflect the same degree of adiposity or intra-abdominal pressure as observed in high-income settings. Instead, it may also capture differences in body composition, parity, or lifestyle factors. Nevertheless, the association observed in our study suggests that even modest increases in BMI may contribute to recurrence risk in this population.

Women who suffered postoperative complications like hematoma in the pararectal space and postoperative vaginal cuff infection were more likely to suffer apical recurrence. This is similar to what was reported by Nieminen et al. (2003), where infection in the pararectal space was the most important risk factor for recurrence after sacrospinous fixation. Infection in the pararectal space causes weakening of the fascial tissues and the ligament/muscle complex, leading to displacement of the fixation sutures, hence recurrence. A similar mechanism occurs with a hematoma in the pararectal space.

In this study, women who had a vaginal hysterectomy were less likely to suffer recurrence than those in whom the uterus was spared (sacrospinous hysteropexy). This is similar to what was reported by Dietz V et al. [26]. However, Schulten (2022) found that vaginal hysterectomy with vault fixation was more of a greater risk factor for recurrence than sacrospinous hysteropexy [20]. Our contrasting findings could be explained by differences in patient characteristics. Women who underwent sacrospinous hysteropexy were generally younger, and age is an important factor in recurrence, as younger women are more likely to experience recurrence after surgery [25,30]. In our low-resource setting, younger women are also more likely to resume physically strenuous work after surgery, which may increase intra-abdominal pressure and contribute to failure of fixation sutures [5]. In addition, many women in our cohort presented with advanced and longstanding prolapse, often with prolonged cervical exposure. This may result in tissue changes such as keratinization, edema, ulceration, or fibrosis, which can compromise tissue integrity and reduce the ability of the cervix to securely hold fixation sutures, thereby increasing the risk of suture pull-through following sacrospinous hysteropexy [26]. However, the magnitude of the observed protective effect should be interpreted with caution. Differences in baseline characteristics between groups, particularly age and postoperative activity levels, may have influenced the observed association. Furthermore, the relatively small number of women undergoing hysteropexy may have limited the precision of the estimate, and residual confounding cannot be excluded. While hysteropexy remains valuable for uterine preservation, careful patient selection and counseling are essential in low-resource settings.

 Our study has several strengths. To the best of our knowledge, it is among the first to evaluate recurrence of apical prolapse after sacrospinous ligament fixation in a low-resource setting where this procedure is commonly performed. In addition, data were collected prospectively, and recurrence was assessed using the standardized Pelvic Organ Prolapse Quantification (POP-Q) system validated by the International Continence Society. However, some limitations should be considered. The relatively short follow-up period of one year may underestimate long-term recurrence, and studies with longer follow-up are recommended. Furthermore, this was a single-center study, which may limit generalizability to other settings. Finally, postoperative complications were limited to hematoma and vaginal cuff infection; other relevant complications, such as buttock pain, nerve injury, urinary retention, ureteral injury, and de novo stress urinary incontinence, were not systematically assessed, which may have led to underestimation of postoperative morbidity and could influence the observed association between complications and recurrence.

## Conclusions

Recurrence of apical prolapse after sacrospinous ligament fixation is common, occurring in about two in every 10 women, particularly among the obese/overweight and those who suffer postoperative complications. Vaginal hysterectomy provides a protective effect against recurrence. We recommend weight optimization strategies, especially for overweight/obese women, enhanced perioperative care to minimize postoperative complications, and considering vaginal hysterectomy in uterine prolapse, where uterine sparing surgery is not required.
